# Hyperglycemia Enhances the Proliferation of Non-Tumorigenic and Malignant Mammary Epithelial Cells through Increased leptin/IGF1R Signaling and Activation of AKT/mTOR

**DOI:** 10.1371/journal.pone.0079708

**Published:** 2013-11-18

**Authors:** Rebecca Lopez, Arunkumar Arumugam, Riya Joseph, Kanika Monga, Thiyagarajan Boopalan, Pamela Agullo, Christina Gutierrez, Sushmita Nandy, Ramadevi Subramani, Jose Manuel de la Rosa, Rajkumar Lakshmanaswamy

**Affiliations:** Center of Excellence in Cancer Research, Center of Excellence in Diabetes Research, Department of Biomedical Sciences MSB1, Texas Tech University Health Sciences Center, Paul L. Foster School of Medicine, El Paso, Texas, United States of America; University of Pittsburgh Cancer Institute, United States of America

## Abstract

Obesity and diabetes are associated with increased breast cancer risk and worse disease progression once cancer is diagnosed; however, the exact etiology behind these observations remains to be fully elucidated. Due to the global obesity/diabetes pandemic, it is imperative to understand how these diseases promote and enhance breast cancer and other common cancers. In this study we demonstrate that hyperglycemia promotes breast cancer by altering leptin/IGF1R and AKT/mTOR signaling. To our knowledge, we show for the first time that in breast epithelial cells, hyperglycemia alone directly impacts leptin signaling. Hyperglycemia increased proliferation of both non-tumorigenic and malignant mammary epithelial cells. These observations coincided with increased leptin receptor and IGF1R receptor, as well as, increased levels of GRB2, pJAK2, pSTAT3, pIRS1/2, pAKT, and p-mTOR. Moreover, pJAK2 was almost completely colocalized with leptin receptor under high glucose conditions. These results demonstrate how hyperglycemia can potentially increase the risk of breast cancer in premalignant lesions and enhance cancer progression in malignant cells.

## Introduction

Breast cancer is the leading cause of cancer death among women worldwide [1], [2]. It is now well-established that diabetes increases the risk of various cancers including breast, liver, pancreatic, bladder, colorectal, non-Hodgkin's lymphoma, and endometrial cancer [3]. According to recent studies, diabetes conferred as much as 37% increased risk of breast cancer in women [4] and both diabetes and breast cancer incidence are increasing at alarming rates worldwide [3]. Even in the absence of overt diabetes, both prediabetes and metabolic syndrome may also increase the risk of certain cancers, including breast cancer [5]–[8]. A great deal of work has shown that cancerous cells become highly dependent on glucose and grow best in media containing high-glucose concentrations [7], [8]. Thus, it is not surprising that diabetes is also associated with worse disease progression once cancer is diagnosed [3].

Likewise, obesity increases the risk and severity of certain cancers including breast cancer [3]. Overweight/obesity often precedes or accompanies the development of diabetes and the global obesity epidemic continues to worsen in both adults and children [3]. In recent studies, obesity increased the risk of postmenopausal breast cancer by as much as 55% [4]. Based on a growing body of evidence, multiple factors likely contribute to the increased incidence and severity of breast cancer in overweight/obese individuals. These include increased hormone production, increased leptin signaling, and increased growth factor signaling (insulin/IGF1), as well as, decreased adiponectin signaling and decreased production of IGFBPs (insulin-like growth factor binding proteins) and SHBGs (sex hormone binding globulins) [3], [9]. However, the exact molecular mechanisms by which hyperglycemia and obesity enhance the development and progression of breast cancer remain to be fully elucidated.

Here, we demonstrate that hyperglycemia clearly increases proliferation of both non-tumorigenic and malignant mammary epithelial cells and this is accomplished by increased leptin signaling and pro-survival AKT/mTOR signaling. To our knowledge, this is the first study demonstrating that hyperglycemia alone directly enhances leptin signaling in non-tumorigenic and malignant mammary epithelial cells. This represents at least one mechanism by which diabetes results in worse cancer progression. Moreover, relative to malignant mammary epithelial cells, non-tumorigenic mammary epithelial cells derived the greatest growth benefit from hyperglycemia. All together, these results demonstrate that hyperglycemia alone enhances the growth of non-tumorigenic breast epithelial cells, as well as, malignant breast epithelial cells. This could be the reason for increased risk of breast cancer in normal tissue and one of the reasons for enhanced breast cancer progression in malignant lesions.

## Materials and Methods

### Cells and cell culture

Triple negative non-tumorigenic MCF10A cells, triple negative MDA-231 cells, and hormone-receptor positive MCF7 cells were obtained from ATCC and were maintained according to the ATCC cell culture guidelines in media containing normal physiological glucose levels (5 mM). In order to mimic diabetic levels of glucose in downstream experiments, cells were transitioned into media formulated with 10 mM glucose (HG, high glucose), as required. We chose 10 mM glucose for hyperglycemia studies because this represents a physiologically relevant concentration of glucose commonly encountered in diabetic individuals [10].

### Cell proliferation

Cell proliferation in normal and high glucose was assessed via MTS assay. For this experiment we chose to assess two levels of high glucose (10 mM and 25 mM) to determine whether hyperglycemia has a dose-dependent effect on cell proliferation. For each condition, cells were seeded in triplicate in 24-well plates. Cells were seeded in 500 µL of media per well and spent media was aspirated and replaced in each well every 24 hr. Cell proliferation was measured at 24 hr, 48 hr, and 72 hr. Cells were incubated with MTS reagent at 37°C for 4 hr and absorbance at 490 nm was measured using the Victor X4 Multilabel Plate Reader (PerkinElmer).

### Apoptosis and cell death

Cell apoptosis and cell death in normal and high glucose was assessed via Annexin V and propidium iodide staining. Cells were seeded into 24-well plates in normal and high glucose. Apoptosis and cell death were then measured at 24 hr, 48 hr, and 72 hr via flow cytometric analysis on the BD AccuriC6 Flow Cytometer (Becton Dickinson). Cells were stained with Annexin V and propidium iodide according to the manufacturer's specifications using the FITC Annexin V Apoptosis Detection Kit I from BD Biosciences. Cells were analyzed immediately after staining.

### Immunoblotting

Cells were seeded into 75 cm^2^ flasks in normal or high glucose. Cells were harvested and whole-cell protein lysates were generated at 24 hr, 48 hr, and 72 hr. Protein concentration was measured using the Pierce BCA (bicinchoninic acid) protein assay (Thermo Scientific) according to the manufacturer's specifications. Clarified cell lysates were aliquoted and stored at −80°C. Equivalent amounts of protein (10 – 20 µg) were resolved via reducing SDS-PAGE using 4–20% gradient pre-cast Mini-Protean TGX polyacrylamide gels (Bio-Rad). Resolved proteins were transferred onto PVDF immunoblotting membranes and probed with the following antibodies as per manufacturer's instructions:

OB-R, JAK2, p-JAK2 (Tyr1007), mTOR, p-mTOR (Ser2448) (Sigma-Aldrich); Leptin (R&D Systems); AKT, p-AKT (Ser473), IGF1R, p-IGF1R (Tyr1135), STAT3, p-STAT3 (Tyr705, Ser727), IRS1, p-IRS1 (Ser636/639), and GRB2 (Cell Signaling); AgRP, IRS2, and p-IRS2 (Ser731) (Abcam); CDK2, Cyclin D1, and IGF1 (Santa Cruz).

For phosphorylated signaling intermediates, blots were first probed for the phosphorylated protein, then stripped and reprobed for the total form of the protein. Each membrane was also stripped and reprobed for β-actin (Sigma-Aldrich) to control for protein loading. In several experiments, membranes were reprobed for several proteins and in these cases the same β-actin loading control is shown. Only proteins with sufficient separation by molecular weight were reprobed from the same membrane. For chemiluminescent detection of proteins, SuperSignal West Femto Chemiluminescent Substrate (ThermoScientific) and SuperSignal West Pico Chemiluminescent Substrate (ThermoScientific) were used according to the manufacturer's instructions. Immunoblots were processed digitally on the ImageQuant LAS4000 biomolecular imager (GE Healthcare).

### Immunofluorescence

MCF10A and MDA-231 cells were seeded onto glass coverslips in 6-well plates in normal or high glucose. At 24 hr, cells were fixed with methanol, washed, permeabilized, and stained with the following antibodies: OB-R (Sigma), p-JAK2 (Sigma), and p-STAT3 (Ser727 and Tyr705) (Cell Signaling). Fluorescence images were captured on the Nikon eclipse Ti6 confocal fluorescence microscope.

### Statistical analysis

Where applicable, statistical significance between groups was calculated using Student's t-test. Differences with a p-value ≤0.05 were considered significant. Where applicable, data are represented as mean±SEM.

## Results

### Cell proliferation under hyperglycemia

**Figure 1:**
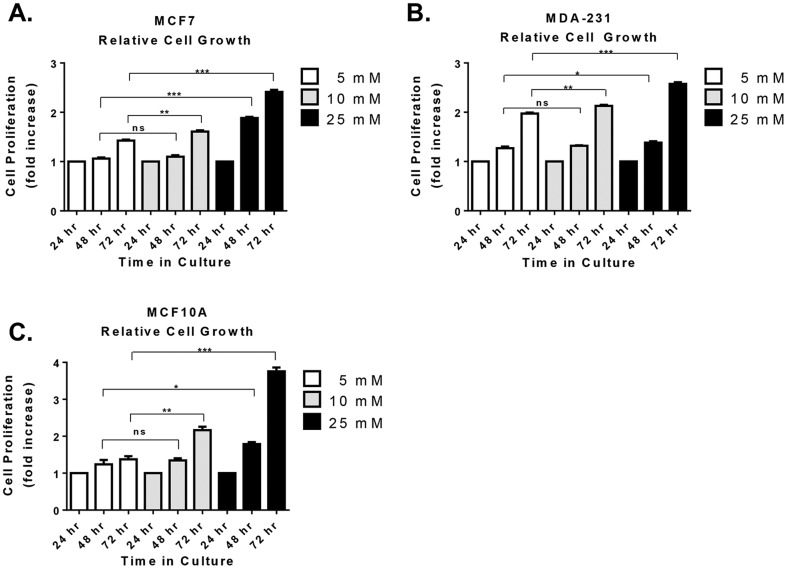
Cell proliferation under hyperglycemia. Cells were cultured in three concentrations of glucose including 5(normal) and 10 and 25 mM (high glucose). Changes in cell proliferation were assessed via MTS assay at 24, 48, and 72 hr in estrogen-receptor positive breast cancer cells (A), triple negative breast cancer cells (B), and non-tumorigenic breast epithelial cells (C). In each case, results were first normalized to baseline growth at 24 hr for each condition and expressed as fold change in cell proliferation above the 24 hr baseline. Results shown here are representative of at least five separate experiments. Statistical significance was calculated using unpaired Student's t-test. Results are expressed as mean±SEM. ns, not statistically significant *, p-value≤0.05 **, p-value≤0.01 ***, p-value≤0.001.

*In vitro* cell proliferation in normal and high glucose was measured by MTS assay in non-tumorigenic breast epithelial cells (MCF10A), estrogen/progesterone-receptor positive breast cancer cells (MCF7), and triple negative breast cancer cells (MDA-231). Compared to normal glucose, high glucose (10 and 25 mM) resulted in increased cell proliferation within 72 hr in both non-tumorigenic and malignant breast epithelial cells (Figure 1). It is interesting to note that under normal glucose conditions, MDA-231 cells exhibited the most robust growth rate of all the three cell lines tested (Figure 1B). Yet in high glucose MCF10A cells exhibited cell growth just as robust as that for MDA-231 cells (Figure 1C), indicating a greater rate of growth in MCF10A cells under hyperglycemia. Therefore, hyperglycemia particularly supports or enhances the growth of non-tumorigenic triple negative breast epithelial cells, which potentially translates to an increased risk of breast cancer in normal tissue or in premalignant lesions. Moreover, glucose appears to have a dose-dependent effect on cell proliferation because cell proliferation was further enhanced in 25 mM glucose compared to 10 mM glucose for all three cell lines.

### Apoptosis and cell death under hyperglycemia

**Figure 2:**
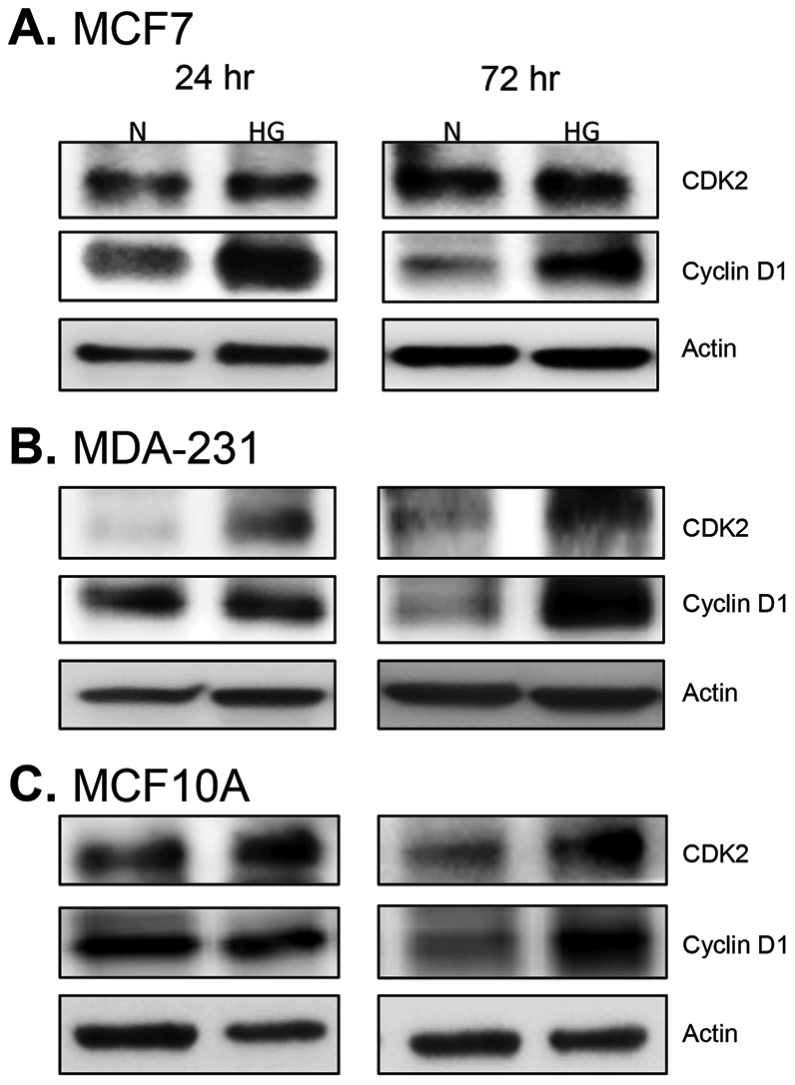
Cell cycle proteins increase with hyperglycemia. Cells were cultured 24 and 72(N) and high glucose (HG). Levels of CDK2 and Cyclin D1, which enhance progression through the G1 and S phases of the cell cycle, were assessed by Western blot. Levels of these proteins were assessed in estrogen-receptor positive breast cancer cells (A), triple-negative breast cancer cells (B), and non-tumorigenic breast epithelial cells (C). Ten micrograms of protein was loaded in each lane. Results shown are representative of two separate experiments.

In order to determine whether the observed increase in cell proliferation was a result of differences in cell death and apoptosis, Annexin V and propidium iodide incorporation was measured. Compared to normal glucose, there were no significant differences in early or late apoptosis in high glucose for any of the cell lines tested (data not shown). Likewise, despite minor increases in cell death under high glucose conditions, there were no remarkable changes in cell death (data not shown), Overall, levels of apoptosis and cell death were very low for all cell lines in both normal and high glucose (well below 8% in most cases). Therefore, increasing levels of glucose generally favor cell proliferation and not cell death. This is in agreement with previous studies demonstrating that hyperglycemia induces increased cell cycle progression and DNA synthesis in breast cancer cells [11]–[13]. Indeed, similar to previous observations [12], we also observed a significant increase in cell cycle-associated proteins CDK2 and Cyclin D1 in both non-tumorigenic and malignant cells (Figure 2). This indicates that hyperglycemia increases cell proliferation through enhanced cell cycle progression in both non-tumorigenic breast epithelial cells and malignant breast epithelial cells.

### Leptin receptor and IGF1R signaling under hyperglycemia

**Figure 3:**
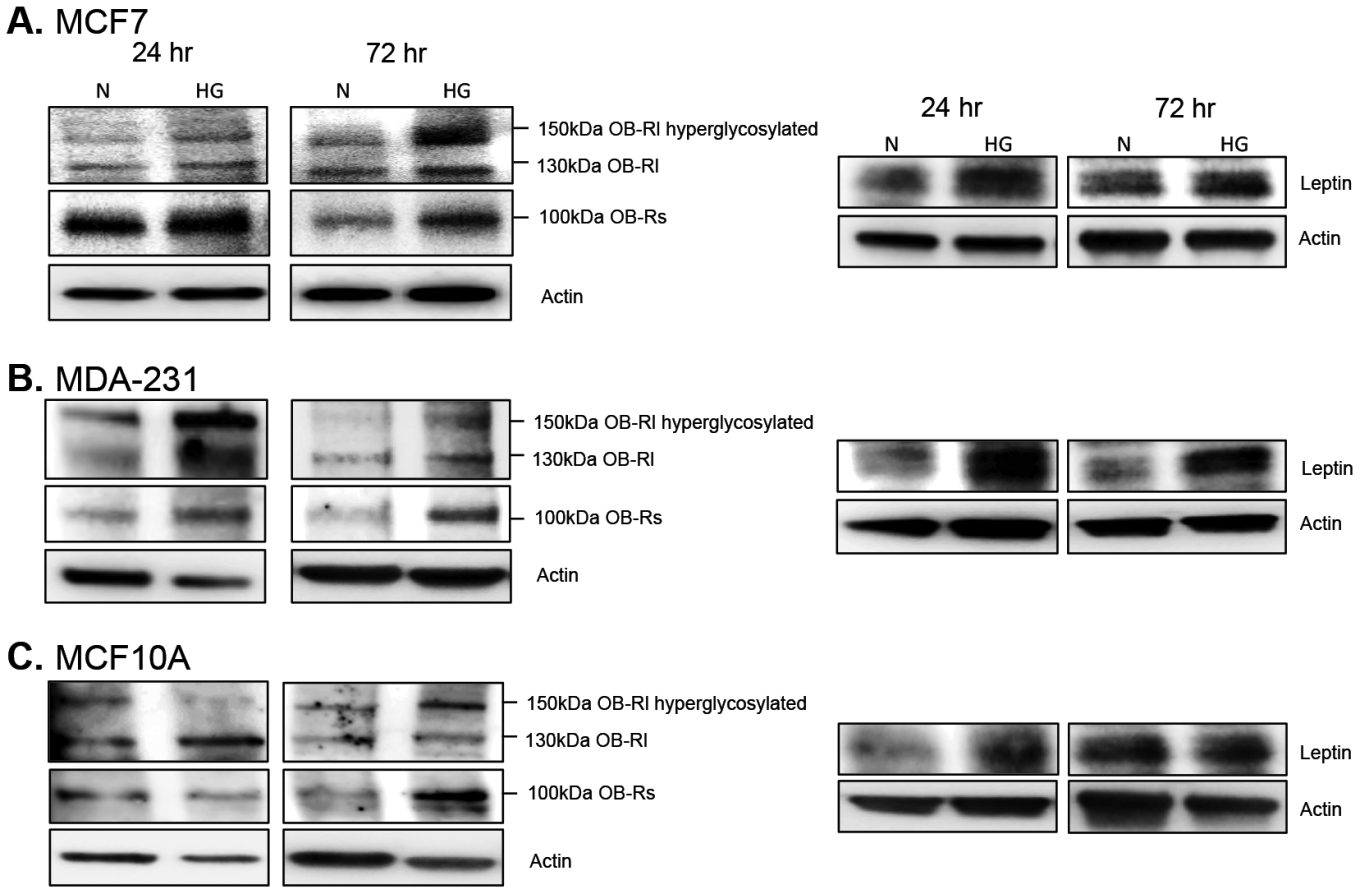
Leptin receptor expression. Cells were cultured in normal (N) and high glucose (HG) for 24 and 72 hr. Changes in leptin receptor expression were assessed via Western blot for estrogen-receptor positive breast cancer cells (A), triple-negative breast cancer cells (B), and non-tumorigenic breast epithelial cells (C). A total of 10 µg of protein was loaded for each sample. Results are representative of at least three separate experiments. ‘OB-Rs’ is the short isoform of leptin receptor, ‘OB-Rl’ is the long isoform of leptin receptor.

**Figure 4:**
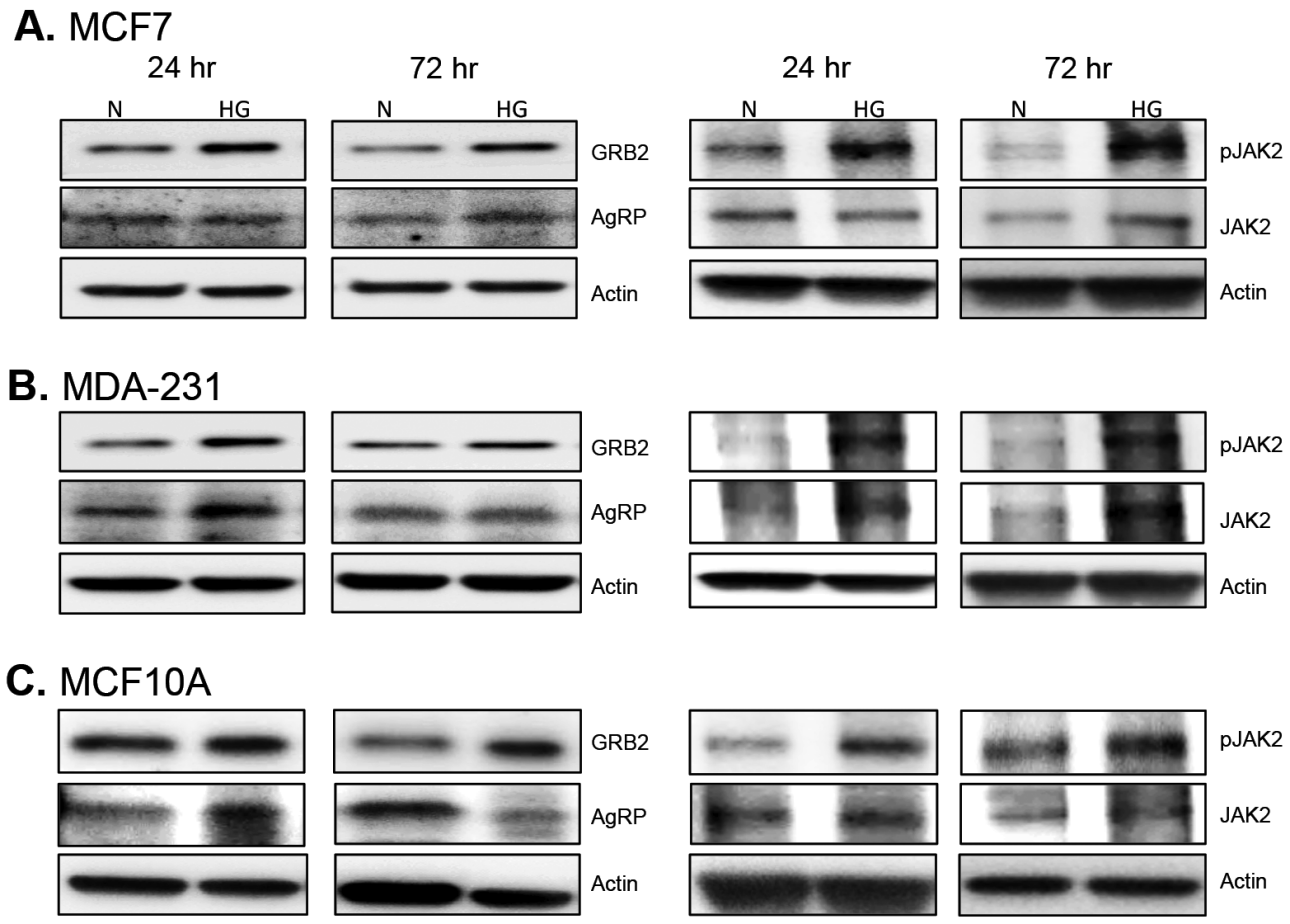
GRB2, AgRP, and JAK2 expression. Cells were cultured in 5(N) and 10 mM (HG) glucose for 24and 72 hr. Changes in GRB2, AgRP, and JAK2 (total and active) protein levels were assessed via Western blot for estrogen-receptor positive breast cancer cells (A), triple-negative breast cancer cells (B), and non-tumorigenic breast epithelial cells (C). A total of 10 µg of protein was loaded for each sample and results are representative of at least three separate experiments.

Due to the direct connection between obesity and type 2 diabetes, molecular targets that often link these two diseases were assessed beginning with leptin receptor signaling (Figure 3). All three of the most abundant leptin receptor isoforms were upregulated by high glucose (Figure 3), indicating an increase in leptin receptor signaling. The expression of GRB2 (growth factor receptor-bound protein 2), a key adapter protein required for transmission of leptin, IGF1R and insulin receptor signals, was also upregulated by high glucose levels (Figure 4). In addition, AgRP (Agouti related protein), which is regulated by leptin, and together with leptin, balances satiety signals in the body [14], was differentially expressed under normal vs. high glucose conditions. In non-tumorigenic and malignant triple negative cells, high glucose enhanced AgRP levels at 24 hr, which subsequently declined to baseline or below baseline levels within 72 hr (Figure 4B, C). In hormone-receptor positive cells, AgRP levels increased at 72 hr under high glucose conditions (Figure 4A). Finally, hyperglycemia also enhanced endogenous expression of leptin protein in all three cell lines (Figure 3).

**Figure 5:**
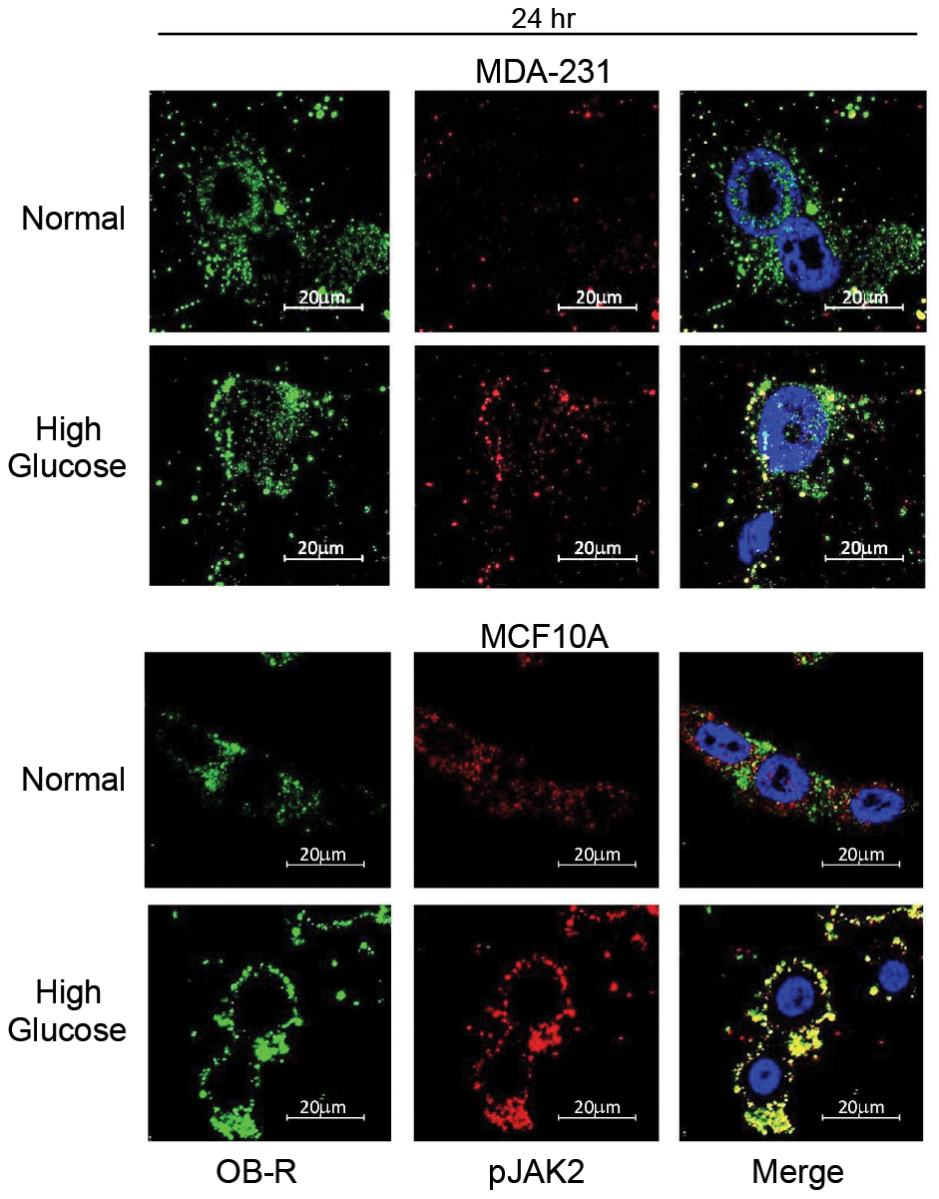
Expression pattern of leptin receptor and JAK2. MDA-231 and MCF10A cells were cultured in normal glucose (5 mM) or high glucose (10 mM) for 24 hr and then subjected to immunofluorescence staining for leptin receptor (left panel, green) and active JAK2 (middle panel, red). Images merged with DAPI reveal the level of colocalization between leptin receptor and JAK2 within individual cells (right panel).

**Figure 6:**
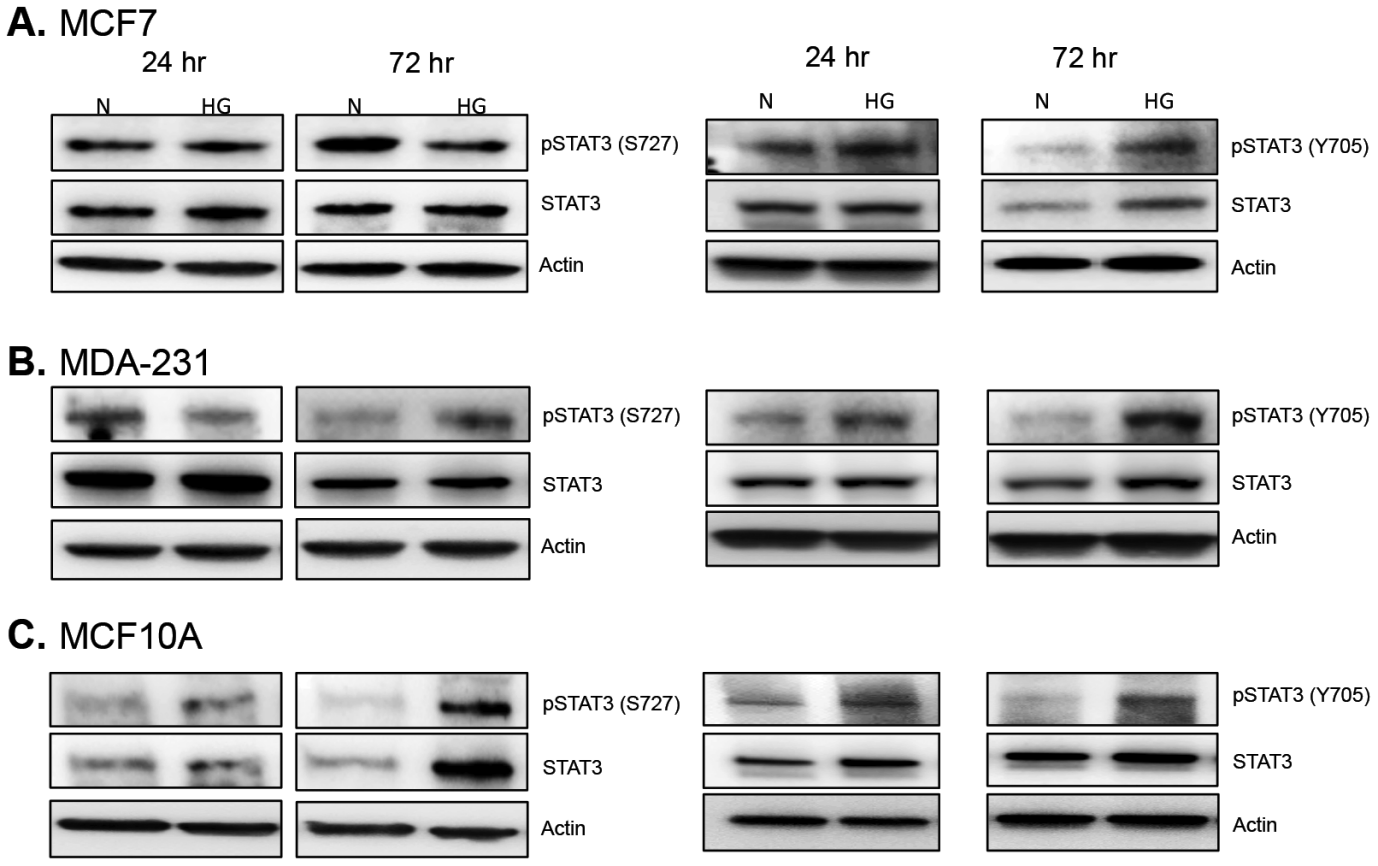
Expression and activation of STAT3. Cells were cultured in normal (N) and high glucose (HG) levels for 24 and 72 hr. Changes in the phosphorylated and total forms of STAT3 were detected via Western blot in MCF7 cells (A), MDA-231 cells (B), and non-tumorigenic MCF10A cells (C). Fifteen micrograms of protein was loaded for each sample. Data shown is representative of at least three separate experiments.

**Figure 7:**
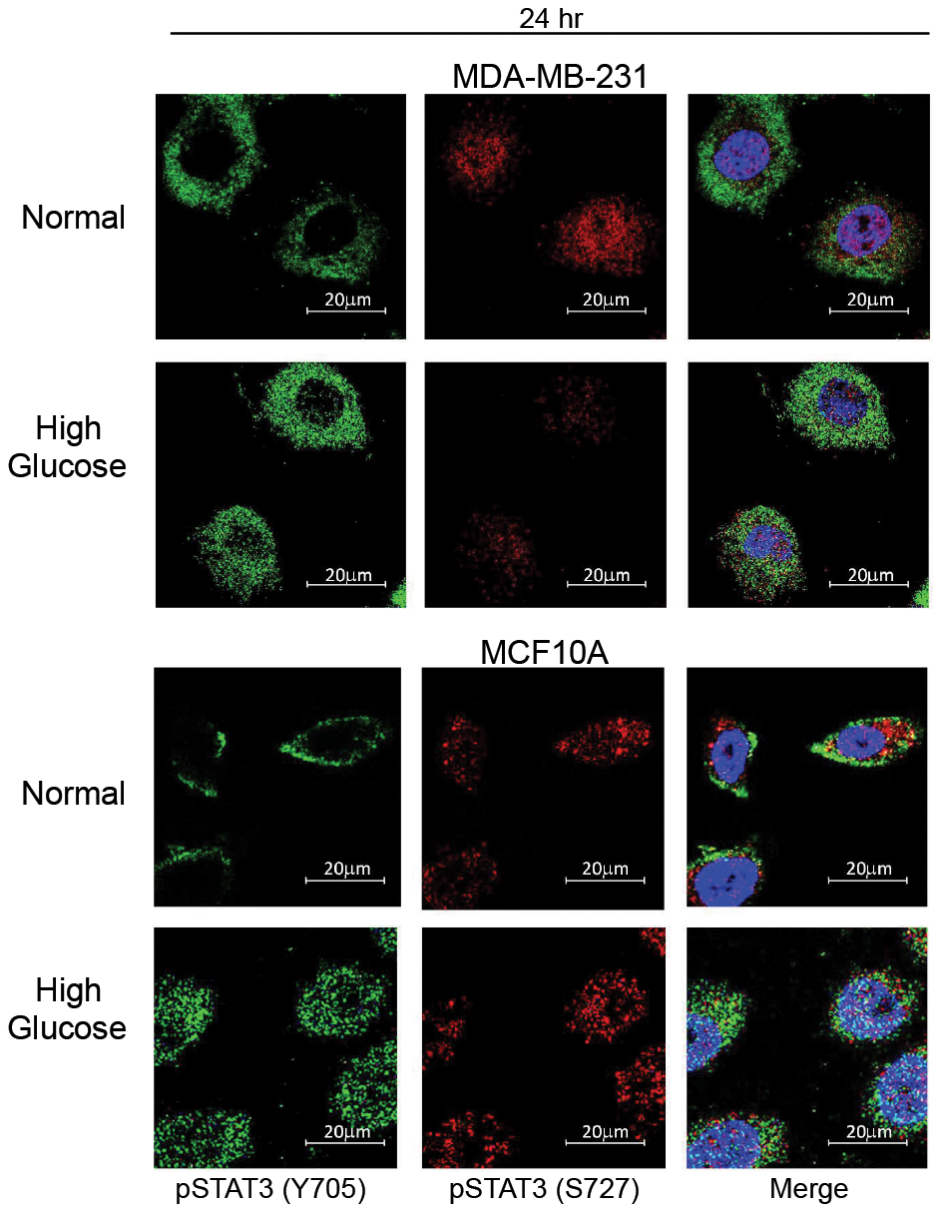
Localization of active STAT3. MDA-231 cells and MCF10A cells were cultured in normal (5 mM) or high glucose (10 mM) for 24 hr. The levels and cellular localization of active STAT3 phosphorylated at two distinct sites were then assessed via immunofluorescence staining. Levels of STAT3 phosphorylated at tyrosine 705 are shown in the left panel (green) and levels of STAT3 phosphorylated at serine 727 are shown in the middle panel (red). The two images were then merged with DAPI to distinguish cytosolic and nuclear distribution.

Leptin receptor signaling classically utilizes the JAK2/STAT3 signaling pathway; therefore, we assessed the levels of JAK2 and STAT3 activation under high glucose conditions and found that hyperglycemia resulted in increased JAK2 phosphorylation in all cell lines within 72 hr (Figure 4). Even more impressive was the finding that within 24 hr activated JAK2 was found almost completely colocalized with leptin receptor in both non-tumorigenic and malignant triple negative cells under high glucose conditions (Figure 5). Differential regulation of STAT3 phosphorylation was also observed with notable differences in the phosphorylation of serine and tyrosine residues (Figure 6, 7). In hormone-receptor positive MCF7 cells, activation of serine 727 remained unchanged at 24 hr and was downregulated at 72 hr (Figure 6A). In contrast, tyrosine 705 phosphorylation was increased at both 24 and 72 hr in MCF7 cells under hyperglycemia (Figure 6A).

In non-tumorigenic and malignant triple negative cells, phosphorylation of both serine 727 and tyrosine 705 was increased with hyperglycemia (Figure 6B, C). Moreover, nuclear localization of phospho-STAT3 (Tyr 705) was increased in both of these cell lines under high glucose conditions (Figure 7). However, nuclear localization of serine 727 phospho-STAT3 appeared to be constitutive in both cell lines regardless of glucose levels (Figure 7). These results suggest that hyperglycemia may primarily modulate STAT3 Tyr705 activation.

**Figure 8:**
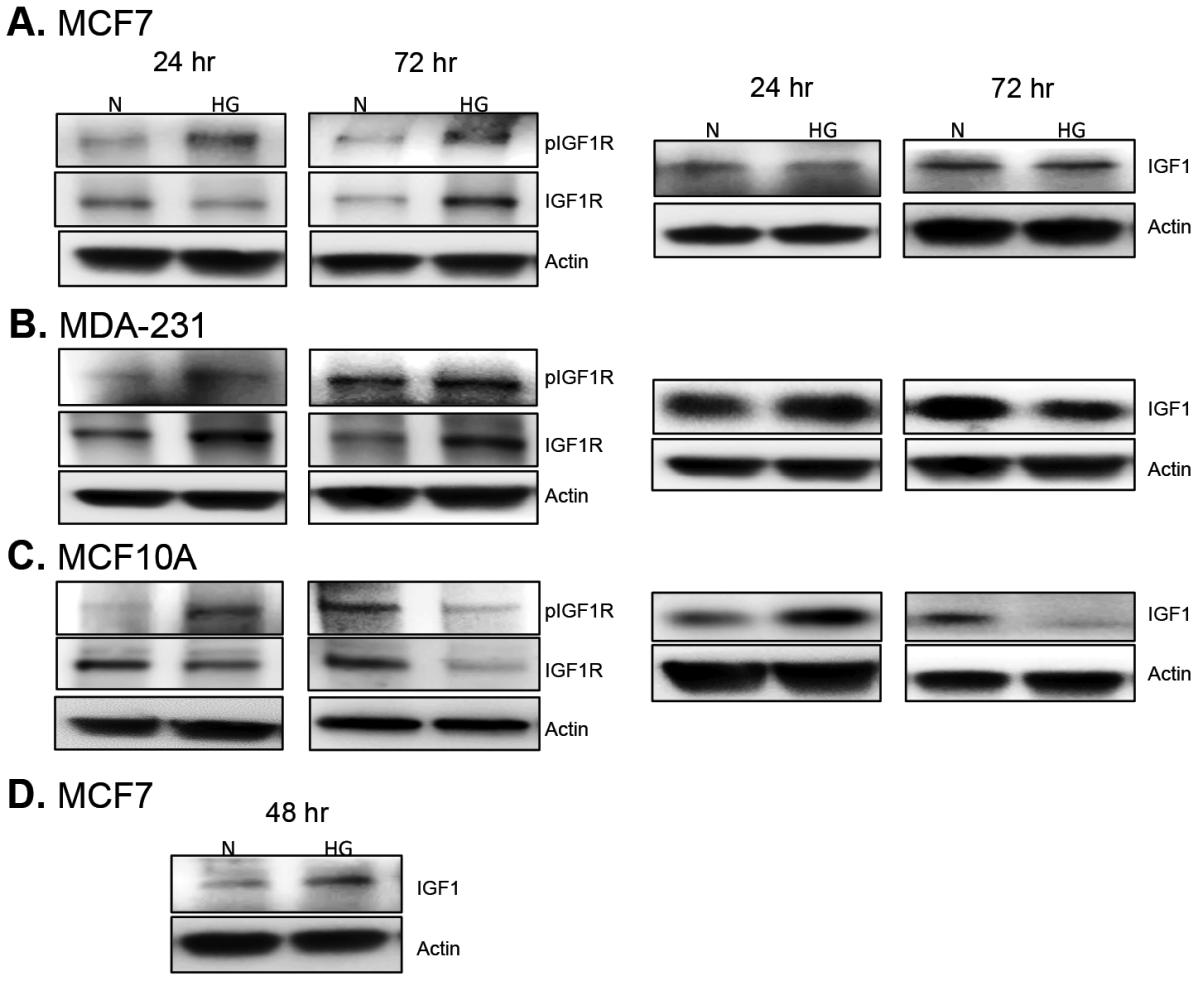
Expression and activation of IGF1R/IGF-1 signaling. Cells were cultured in normal (N) and high glucose (HG) for 24 and 72 hr. Using Western blot, changes in the phosphorylated and total form of IGF1R were determined for normal and diabetic conditions (left panel). Likewise, differences in IGF1 production in normoglycemia vs. hyperglycemia were assessed via Western blot (right panel, D). At least three separate experiments were performed and representative images are shown here.

Since there is significant cross-talk between leptin receptor, insulin, and IGF1R/IGF1 (insulin-like growth factor) signaling pathways, we assessed the effect of hyperglycemia on key intermediates within each of these pathways. First, we assessed the levels of active IGF1R, as well as, levels of IGF1. We found that, as expected, IGF1R phosphorylation is increased in cells exposed to high glucose (Figure 8). Interestingly, MCF7 and MDA-231 cells exhibited a more sustained activation of IGF1R with increased phosphorylation observed at both 24 hr and 72 hr (Figure 8A, 8B). IGF1 levels were a little more variable with increased IGF1 expression observed only at 24 hr in MDA-231 cells (Figure 8B) and only at 48 hr in MCF7 cells (Figure 8D). In contrast, non-tumorigenic MCF10A cells demonstrated a more transient activation of IGF1R (Figure 8C). Increased IGF1R phosphorylation was only observed at 24 hr (Figure 8C). Meanwhile, the levels of total IGF1R protein were decreased by high glucose at 24 hr and 72 hr indicating that the only contribution from IGF1R signaling in MCF10A cells is a transient increase in receptor activation at 24 hr (Figure 8C). Likewise, IGF1 levels in MCF10A cells were only increased at 24 hr in high glucose (Figure 8C).

**Figure 9:**
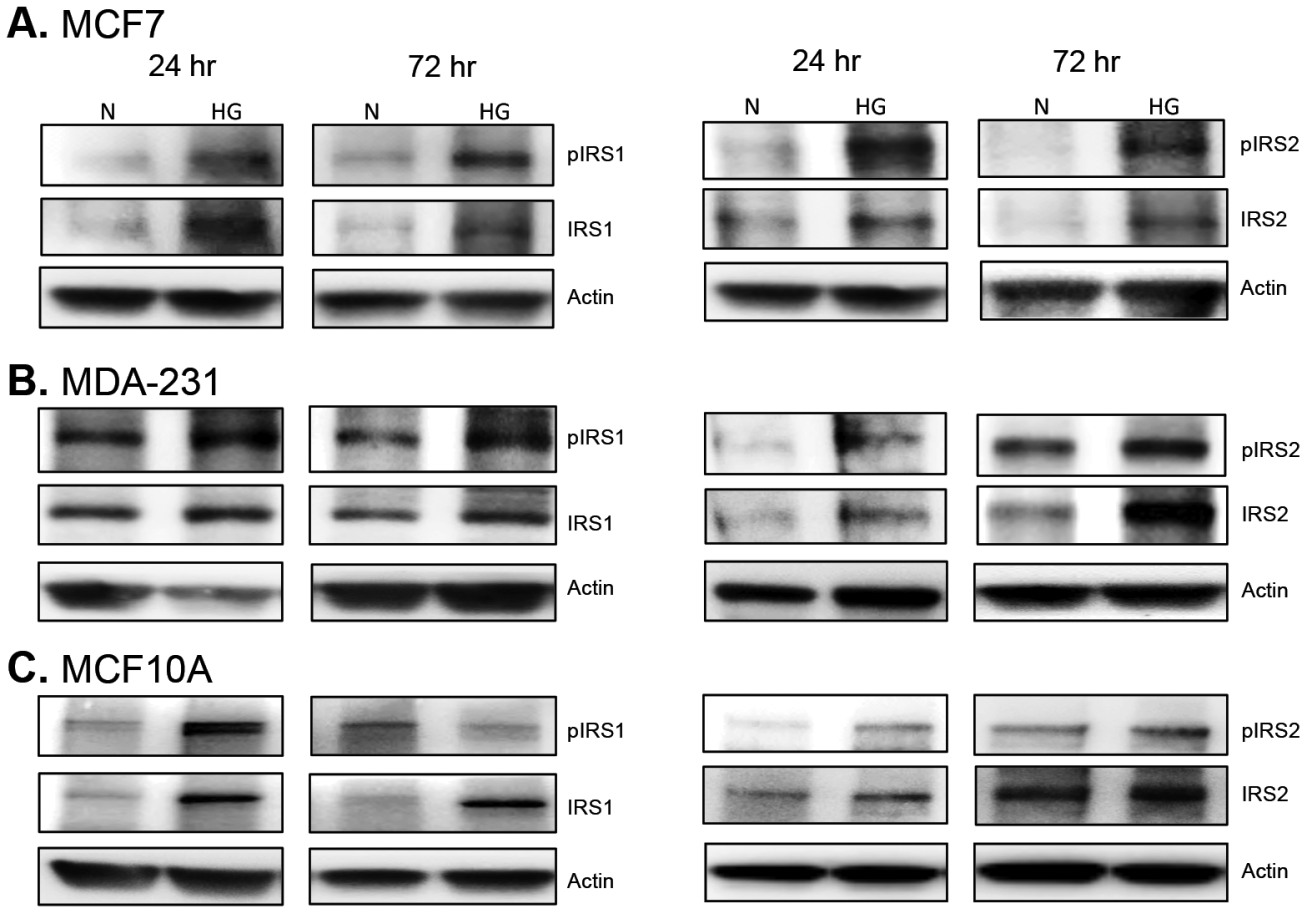
Expression and activation of IRS1/IRS2. MCF7 (A), MDA-231 (B), and MCF10A cells (C) were cultured in 5 mM (N) or 10 mM glucose (HG). After 24 and 72 hr, cells were harvested and whole cell lysates were subjected to Western blot analysis for IRS1 (left panel) and IRS2 (right panel). A total of 10 µg of protein was loaded per sample and results shown are representative of three replicate experiments.

We next determined the levels of active IRS1 and IRS2 (insulin receptor substrate proteins), which are critical for mediating both insulin receptor and IGF1R signaling. Under hyperglycemia, we found sustained phosphorylation of IRS2 in all three cell lines (Figure 9). IRS1 phosphorylation was also sustained in malignant cells (Figure 9A, B), but was only increased at 24 hr in MCF10A cells (Figure 9C). Therefore, as expected, hyperglycemia enhances IGF1R/insulin receptor signaling in both non-tumorigenic and malignant breast epithelial cells.

### AKT/mTOR signaling under hyperglycemia

**Figure 10:**
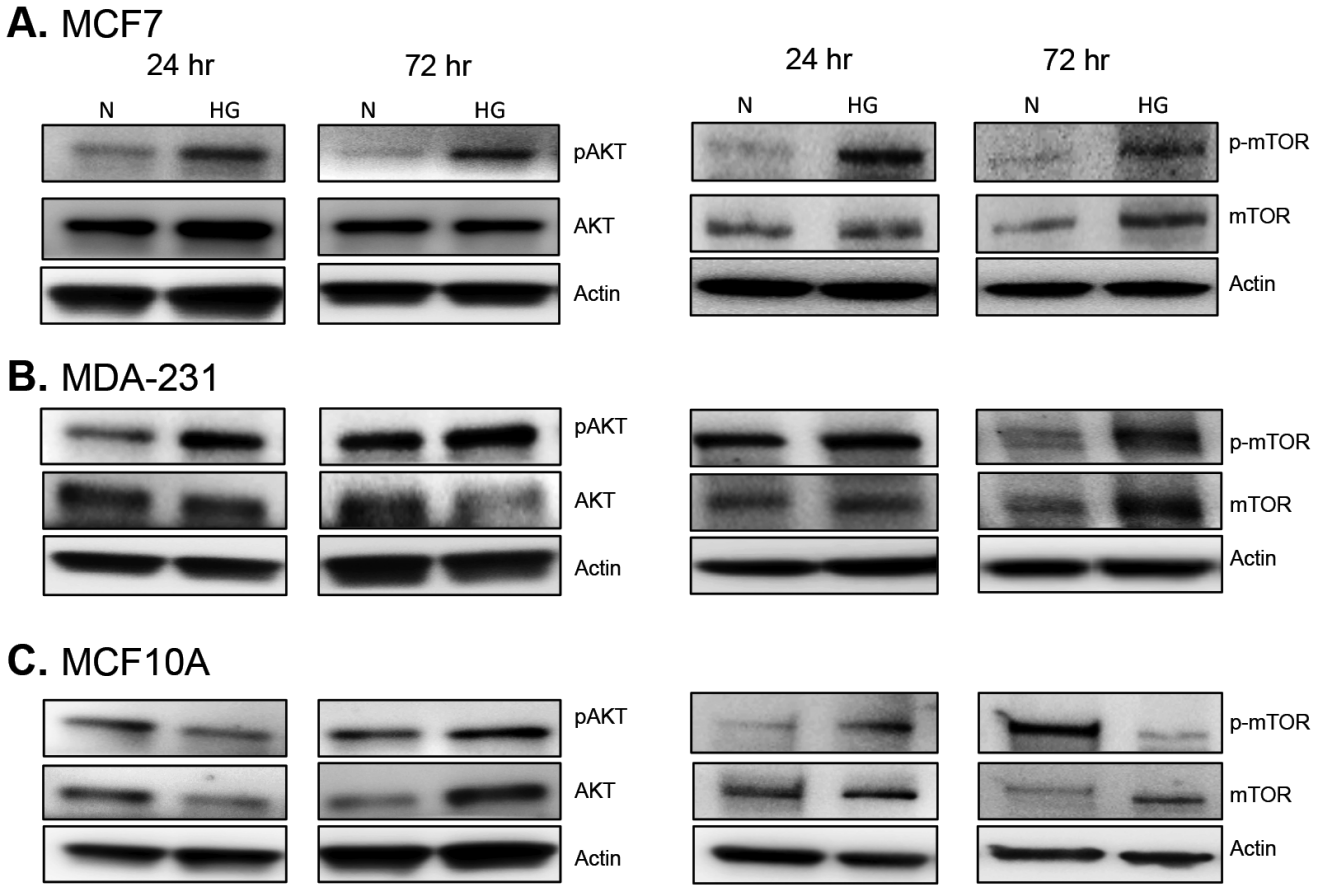
Expression and activation of AKT and mTOR. Changes in the phosphorylated and total form of AKT (left panel) and mTOR (right panel) were assessed via Western blot in estrogen-receptor positive MCF7 cells (A), triple-negative MDA-231 cells (B), and non-tumorigenic MCF10A cells (C) exposed to either normal (5 mM) or high glucose (10 mM) levels. Twenty micrograms of total protein was loaded for each sample and changes in protein levels were determined at 24 hr and 72 hr. Results are representative of at least three separate experiments.

In order to determine the effects of hyperglycemia on signaling events further downstream, we assessed activation of AKT/mTOR. AKT and mTOR are key signaling intermediates that are positively regulated by leptin, IGF1R, and insulin signaling. AKT/mTOR activation is known to enhance cell proliferation in the obese/diabetic state [15], [16]. Here, enhanced activation of both AKT and mTOR was observed in all three cell lines under high glucose conditions (Figure 10). Hyperglycemia resulted in activation of AKT and mTOR as early as 24 hr in MCF7 and MDA-231 cells and this effect was sustained through 72 hr (Figure 10A, B). In MCF10A cells, AKT phosphorylation was not increased under hyperglycemia until 72 hr, but mTOR phosphorylation was increased at both 24 hr and 72 hr (Figure 10C).

## Discussion

Current evidence demonstrates a direct link between obesity, diabetes, and increased risk of breast cancer [3]. In addition, there is indisputable evidence that obesity and diabetes result in much worse disease progression in patients with breast cancer [17]. Of even greater concern are recent studies linking metabolic syndrome with increased risk and more severe progression of breast cancer [6]. To date, extensive research has revealed some of the key effects of diabetes and obesity on breast cancer promotion and progression, however, many questions remain. A key hallmark associated with obesity and diabetes, as well as, metabolic syndrome is aberrant glucose metabolism or hyperglycemia [18]. Therefore, we sought to determine how hyperglycemia affects the growth of non-tumorigenic and malignant mammary epithelial cells. In addition, we wanted to elucidate downstream cellular signaling events that take place in non-tumorigenic and malignant mammary epithelial cells as a result of exposure to hyperglycemia.

We show for the first time that hyperglycemia alone directly enhances leptin signaling in both non-tumorigenic and malignant breast epithelial cells. This effect of hyperglycemia on leptin signaling was also recently demonstrated by Su and colleagues in human fibrosarcoma and murine hypothalamic tumor cells [19]. In parallel with previous studies [11]–[13], we further demonstrate that hyperglycemia significantly increases the proliferation of highly aggressive hormone receptor negative breast cancer cells and hormone receptor-positive breast cancer cells. However, hyperglycemia appears to have the most profound impact on non-tumorigenic breast epithelial cells. This abnormally high rate of cell proliferation likely predisposes non-tumorigenic cells to an increased risk of DNA damage and subsequent accumulation of mutations that may ultimately result in malignant transformation. This is one reason why obesity, metabolic syndrome and diabetes may lead to an increased risk of breast cancer. In addition, the observed increase in malignant cell proliferation could also enhance tumor growth and cancer progression once malignant transformation occurs. This helps explain why obesity and diabetes result in worse disease progression in breast cancer patients.

Moreover, while it is well-established that diabetes and obesity increase the risk of hormone-receptor positive breast cancer in postmenopausal women [20]–[22], there is now accumulating evidence that indicates an increased risk of hormone receptor negative breast cancer in women with metabolic syndrome [23] or obesity [20]. Since hyperglycemia is frequently a key hallmark of metabolic syndrome and obesity, the results presented here not only have implications for the etiology of hormone-receptor positive breast cancer, but may also have important implications for the etiology of hormone-receptor negative breast cancer in women with metabolic syndrome or obesity. Here, we show that hyperglycemia has similar protumorigenic effects on triple-negative and hormone-receptor positive breast cancer cells.

Observations from previous studies have shown that hyperglycemia induces an increase in cell cycle progression and DNA synthesis in breast cancer cells [11]–[13]. Therefore, we sought to determine whether hyperglycemia may decrease levels of apoptosis or cell death, but we did not observe any major differences in apoptosis or cell death compared to normal glucose conditions. So, despite increased cell proliferation there is no significant alteration in cell death or apoptosis under hyperglycemia. However, in parallel with previous studies [12], [13], we demonstrate enhanced expression of CDK2 and Cyclin D1 upon exposure to hyperglycemia, indicating an accelerated cell cycle in response to hyperglycemia. Yamamoto and colleagues [13], previously showed that high glucose accelerates cell-cycle progression in MCF7 cells through decreased expression of PKC-betaII. Likewise, Okumura et al. [12] demonstrated that hyperglycemia induced increased CDK2 and Cyclin D1 expression in MCF7 cells and this effect was further enhanced with the addition of exogenous leptin. In fact, treatment with leptin alone resulted in an increase in DNA synthesis which was further enhanced when leptin was given together with high glucose [12]. This effect may have been partially mediated through leptin-induced PKC-alpha, the expression of which was also further increased by a combination of high glucose and leptin treatment [12]. These observations are in line with our own observations in the current study, where we show that high glucose alone is able to enhance endogenous leptin production and also lead to increased leptin receptor signaling in both non-tumorigenic and malignant mammary epithelial cells.

In the current study, increased levels of both long and short forms of leptin receptor, as well as, increased levels of GRB2, phospho-STAT3, and phospho-JAK2 were observed under hyperglycemia. Moreover, active JAK2 and leptin receptor were almost completely colocalized under hyperglycemia providing strong evidence that leptin receptor signaling is directly induced by high glucose levels in mammary epithelial cells. In addition, hyperglycosylated isoforms of the long form of leptin receptor (150 kDa) were particularly favored by hyperglycemia. Likewise, the short isoform of leptin receptor was increased under hyperglycemia. The full-length, transmembrane form of leptin receptor is the form predominantly associated with transmission of leptin receptor signals [24]. However, several studies have demonstrated that there are at least 6 different isoforms of leptin receptor, which include hyperglycosylated isoforms and a truncated short isoform [24]. While the exact function of these additional isoforms remains to be fully elucidated, studies indicate that hyperglycosylation affects the binding affinity of leptin for leptin receptor [24]. Thus, our observation that hyperglycemia upregulates hyperglycosylated forms of leptin receptor indicates that high glucose not only affects receptor levels, but may also affect the binding affinity of the receptor for its ligand. The short isoform of leptin receptor, which is thought to play a role in intracellular leptin turnover [24], is also a secreted form of leptin receptor [7]. This secreted form of leptin receptor is increasingly recognized for its role in regulating systemic leptin signaling [7]. Thus, any therapy aimed at blocking leptin signaling *in vivo* will need to target both membrane-bound and soluble forms of the leptin receptor.

Earlier studies indicate that receptor-binding peptide fragments may be useful for blocking leptin signaling [7] and would be expected to target all isoforms of the leptin receptor. These receptor-binding leptin fragments have been shown to effectively inhibit leptin-induced cell proliferation in both estrogen-receptor-positive and estrogen-receptor-negative breast cancer cells [7]. However, there is a great need for more defined studies of the function of different leptin-receptor isoforms in mammary epithelial cells, as well as, in other cell types. It will be of particular importance to determine the role of hyperglycosylated forms of leptin receptor in breast cancer progression.

Other important observations from this study include enhanced IGF1R signaling and increased AKT/mTOR activation under hyperglycemia in both non-tumorigenic and malignant breast epithelial cells. AKT/mTOR activation is commonly associated with increased leptin and IGF1R signaling. To our knowledge, this is the first study demonstrating that hyperglycemia alone directly enhances leptin signaling in non-tumorigenic and malignant breast epithelial cells. Glucose-mediated enhancement of leptin signaling in breast tissue and other tissues throughout the body represents at least one mechanism by which hyperglycemia may result in worse cancer promotion and progression. Moreover, these results give us an indication of the particularly detrimental effects of combined obesity and diabetes in terms of increased breast cancer risk and poor prognosis once cancer is diagnosed [3], [17], [24]. Aberrant activation of leptin and IGF1 signaling pathways has been shown to result in increased cell proliferation, enhanced epithelial to mesenchymal transition, and greater invasiveness of breast cancer [25], [26]. Moreover, enhanced epithelial to mesenchymal transition appears to occur in an AKT-dependent manner through TGF-beta and beta-catenin signaling [25], [26]. Therefore, determining the signaling intermediates that commonly regulate each of these pathways would be invaluable for designing more effective therapeutic interventions for patients with obesity, diabetes, and breast cancer.

**Figure 11:**
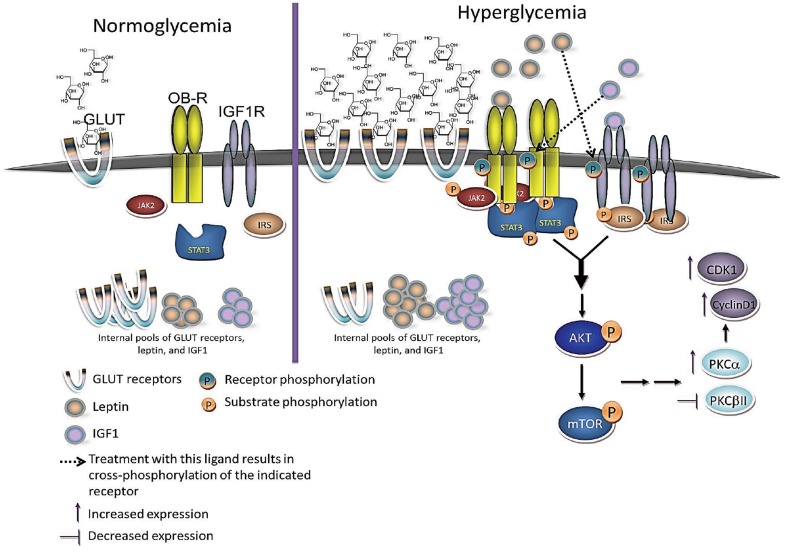
Proposed model of hyperglycemia's protumorigenic effects. Under normal glucose levels or normoglycemia, glucose uptake and leptin/IGF1R signaling remain at relatively low basal levels. Under hyperglycemia, cells respond by increasing glucose uptake through GLUT receptors and this in turn activates metabolic pathways that not only respond to increased glucose levels but also have mitogenic effects on breast epithelial cells. Two key metabolic pathways that can have this effect include the leptin and IGF1R signaling pathways. Breast epithelial cells respond to increased glucose levels by increasing production of leptin and IGF1, which in turn increase the expression and activation of leptin and IGF1 receptors at the cell surface. Together, leptin and IGF1 act synergistically to enhance AKT/mTOR signaling leading to increased cell proliferation, in part, through modulation of PKC proteins and cell cycle-associated proteins.

Based on both our current observations and previously published observations discussed above, we propose the following model to describe the protumorigenic effects of hyperglycemia in breast epithelial cells (Figure 11). We propose that hyperglycemic insult leads to increased glucose uptake, which stimulates increased expression and secretion of leptin and IGF1. Leptin and IGF1 then act in an autocrine and paracrine manner to enhance both leptin and IGF1R signaling in breast epithelial cells. This leads to increased expression of leptin receptor and IGF1R and enhanced signaling through these pathways results in the synergistic activation of AKT/mTOR. It has in fact been shown in MCF7, MDA-231 and MDA-468 breast cancer cells that treatment with leptin can phosphorylate IGF1R and activate downstream signaling while treatment with IGF1 phosphorylates leptin receptor and activates downstream signaling [27]. Co-treatment with these two ligands resulted in synergistic phosphorylation and association of leptin/IGF1 receptors along with enhanced AKT activation [27]. Further downstream of these events, the increased expression of cell cycle-associated proteins such as CDK2 and Cyclin D1 can accelerate progression through the cell cycle thus leading to an aberrant increase in cell proliferation under hyperglycemia. This enhances the potential for malignant transformation and enhances tumor progression in malignantly transformed cells. This model is in agreement with previous epidemiological studies in human populations demonstrating that hyperglycemia actually increases the risk of breast cancer development in women [28] and is also associated with worse overall survival in breast cancer patients [29].

In conclusion, we demonstrate that hyperglycemia alone has profound effects on the molecular circuitry of both non-tumorigenic and malignant mammary epithelial cells. A definite crosstalk between hyperglycemia and leptin signaling exists in breast epithelial cells, as well as, other cells types throughout the body. Consequently, components of this pathway may be useful therapeutic targets for the treatment of diabetes, obesity, and ultimately breast cancer. Future studies will be aimed at determining the exact contributions of both leptin-receptor signaling and additional metabolic, endocrine, and proinflammatory pathways induced by hyperglycemia in normal and malignant breast epithelial cells. Of particular interest is where each of these pathways converge in promoting aberrant cell proliferation, survival, and malignant transformation.
